# Acyclovir‐induced psychiatric and renal adverse effects in a diabetic patient: A case report

**DOI:** 10.1002/ccr3.9310

**Published:** 2024-08-19

**Authors:** Soheil Roshanzamiri, Seyyedeh Azam Mousavizadeh, Sareh Razazzadeh, Shadi Ziaie

**Affiliations:** ^1^ Department of Clinical Pharmacy, School of Pharmacy Shahid Beheshti University of Medical Sciences Tehran Iran

**Keywords:** acute kidney injury, acyclovir, drug‐related side effects and adverse reactions, hallucinations

## Abstract

Acyclovir can cause neurotoxicity and nephrotoxicity, especially in diabetic patients with renal impairment. Consider acyclovir dose adjustment, close monitoring of kidney function, and hemodialysis as a potential therapeutic option in such cases.

## 1 INTRODUCTION

Acyclovir, a synthetic nucleoside analog, is widely utilized for treating infections caused by herpes simplex virus (HSV) and varicella‐zoster virus (VZV). Despite its therapeutic efficacy, acyclovir carries significant risks of psychiatric and renal adverse effects, especially in patients with underlying renal impairment, or those receiving high‐dose regimens.

The antiviral activity of acyclovir primarily targets HSV and VZV, demonstrating greater potency against HSV compared to VZV.^1^, ^2^ Neuropsychiatric manifestations associated with acyclovir use are linked to elevated serum levels of its principal metabolite.

In this report, we present a compelling case of acyclovir‐induced psychiatric and renal adverse effects in a 52‐year‐old woman admitted to the emergency department. This case underscores the critical importance of monitoring renal function and appropriately adjusting dosages in high‐risk patients to mitigate these potential risks.

## 2 CASE PRESENTATION

A 52‐year‐old female with a history of type 2 diabetes mellitus was admitted to Labbafinejad Medical Center in Tehran, Iran. She presented with eye erythema and pre‐orbital edema in her right eye, indicative of Herpes zoster ophthalmicus (HZO). Concurrently, her serum creatinine levels rose significantly from 0.9 to 7.34 mg/dL, accompanied by anuria, lower abdominal pain, nausea, anorexia, and bilious vomiting. These symptoms emerged following a 5‐day intravenous acyclovir treatment (500 mg three times daily) administered at a different facility.

Neurological symptoms such as confusion, dysarthria, and audiovisual hallucinations were noted upon admission. The patient reported seeing small blue figures resembling “Smurfs” climbing up her body and perceived several other people in the room who were digging and excavating the ground. Despite these vivid hallucinations, the patient remained awake and alert and did not require admission to the critical care unit.

The patient's vital signs were stable, and there was no history of immunocompromised conditions or chronic kidney diseases. Initial laboratory investigations confirmed stage 3 acute kidney injury (AKI), in accordance with KDIGO practice guidelines.^3^ The patient was also taking metformin (500 mg three times daily) and atorvastatin (20 mg nightly), with no other medications.

Written informed consent was obtained from the patient for inclusion in this case report, in accordance with the Declaration of Helsinki (DoH).

## 3 WORK‐UP

For prevention of stress‐related mucosal damage (SRMD), the patient received oral pantoprazole (40 mg daily) and intravenous haloperidol (0.5 mg as needed for agitation). A 3‐liter normal saline infusion was administered. Due to a lack of patient consent, a lumbar puncture for CSF analysis was not performed; however, brain MRI results were normal.

Following the ophthalmology consultation, which identified dendritic ulcers and crusted lesions, acyclovir treatment was discontinued. The infectious disease service recommended treatment with artificial tear eye drops (every 3 hours), chloramphenicol eye drops every (6 hours), erythromycin eye ointment (every night), and trifluridine eye drops (every 3 hours) to treat ocular shingles. The pharmacotherapy service suggested delaying the decision on continuing intravenous acyclovir treatment until after a hemodialysis trial had been performed.

Hemodialysis can be beneficial in distinguishing between hallucinations caused by acyclovir toxicity and those due to viral encephalitis, especially when a lumbar puncture is not feasible.^4^

The administration of acyclovir was temporarily suspended, and hemodialysis was initiated 24 hours after the patient's admission via a Shaldon catheter.

Symptomatic remission was fully attained following three successive days of hemodialysis. Prior to the patient's discharge, the Shaldon catheter was removed, and the patient was referred to an ambulatory ophthalmology clinic for additional diagnostic assessment and management. The lab results at admission, after three sessions of dialysis, and before discharge are displayed in Table 1.

**TABLE 1:** Lab results.

Parameter	Admission	After 3 dialysis	Before discharge
Urea (mg/dL)	193	76	35
Creatinine (mg/dL)	7.52	5.12	1.93
Sodium (mmol/L)	137	135	135
Potassium (mmol/L)	4.7	4.3	4
FBS (mg/dL)	115	110	114
Uric acid (mg/dL)	5.9	5.9	6
Calcium (mmol/L)	9.1	9	9

## 4 DISCUSSION AND CONCLUSION

Herpes Zoster, which originates from the reactivation of the VZV (human herpes virus type 3), may be experienced by approximately 30% of the population during their lifespan.^5^, ^6^ HZO results from the reactivation of a latent infection in the trigeminal ganglion, affecting the distal nerves of the ophthalmic division.^7^, ^8^ Ophthalmic complications associated with HZO include keratitis, scleritis, uveitis, glaucoma, optic neuritis, retinal necrosis, and oculomotor palsies, which can arise due to inflammation, direct viral damage, immune responses, or scarring.^3^ While HZO typically presents with a unilateral maculopapular or vesicular rash in a dermatomal distribution, ocular involvement occurs in approximately 50% of HZO cases.^9^ Acyclovir, administered orally, intravenously, or topically, is a standard treatment for the ocular complications of HZO. Intravenous acyclovir doses have typically been 30 mg/kg/day, administered in three divided doses.^10^, ^11^ However, exceeding the maximum solubility of free drugs can lead to crystal nephropathy. Additionally, cases of acute tubular necrosis without crystalluria have been linked to IV acyclovir use. Risk factors such as concomitant use of nephrotoxic drugs, preexisting kidney disease, and dehydration can increase susceptibility to renal dysfunction. Fortunately, most cases of renal dysfunction are transient and resolve following the discontinuation of acyclovir.^12^ Neuropsychiatric symptoms have been associated with elevated serum concentrations of acyclovir's primary metabolite, 9‐carboxymethoxymethylguanine (CMMG). Symptoms include confusion, drowsiness, hyperreflexia, myoclonus, hallucinations, incoherence, and unresponsiveness.^13^ Acyclovir and neuropsychiatric adverse effects is a well‐documented phenomenon, particularly in patients with renal impairment. The neurotoxic effects can mimic the presentation of VZV central nervous system disease, leading to diagnostic dilemmas.^14^

Psychatric symptoms are evident in a case report of a 68‐year‐old Hispanic man with end‐stage renal disease who was diagnosed with cutaneous zoster and was started on acyclovir 4 days prior to admission. His family noted worsening confusion, agitation, speech difficulty, and hallucinations.^14^ Another case report of an 82‐year‐old man with a history of end‐stage renal disease presented with progressively worsening confusion and somnolence for the past 4–5 days.^15^ Similarly, a 69‐year‐old morbidly obese woman presented with mental status changes after she was treated with acyclovir for shingles.^16^ These cases, along with our report, demonstrate that acyclovir‐induced neurotoxicity and psychiatric adverse effects present as acute encephalopathy, making it difficult to distinguish from VZV encephalitis on the basis of clinical presentation alone.

The use of hemodialysis as a diagnostic and therapeutic intervention has been reported in several cases, with hemodialysis patients showing significant improvement after the procedure. This procedure is further supported by a case report discussing the resolution of acyclovir‐associated neurotoxicity with the aid of Bayesian‐informed clearance estimates and another case report discussing reversible brain MRI changes in acyclovir neurotoxicity. These cases suggest that hemodialysis can be an effective strategy for managing acyclovir neurotoxicity in patients with renal impairment.^15^, ^17^ However, more research is needed to establish definitive diagnostic criteria and treatment guidelines for this condition.

It is crucial to maintain a high index of suspicion for acyclovir‐induced neurotoxicity, especially given the overlapping neurological signs with herpes simplex encephalitis. The onset of worsening mental status within 24–48 h of high‐dose intravenous acyclovir in patients with renal impairment should prompt clinicians to measure serum and CSF acyclovir levels. The most effective intervention is the discontinuation or dose adjustment of acyclovir, combined with regular dialysis to facilitate the elimination of acyclovir and its metabolite. Additionally, continuous monitoring of the patient is essential, and intubation should be considered if there is a significant deterioration in the patient's mental state to ensure airway protection.

## AUTHOR CONTRIBUTIONS

**Soheil Roshanzamiri:** Conceptualization; investigation; methodology; writing – original draft; writing – review and editing. **Seyyedeh Azam Mousavizadeh:** Data curation; investigation. **Sareh Razazzadeh:** Data curation; investigation. **Shadi Ziaie:** Methodology; supervision.

## FUNDING INFORMATION

This research received no specific grant from any funding agency in the public, commercial, or not‐for‐profit sectors.

## CONFLICT OF INTEREST STATEMENT

The authors declare that there is no conflict of interest regarding the publication of this manuscript.

## CONSENT

Informed consent was obtained from participant included in the study. The participant was informed about the purpose of the study and their rights to withdraw at any time without any consequences.

## PERMISSION TO REPRODUCE MATERIAL FROM OTHER SOURCES

Permission to reproduce material from other sources was obtained where necessary. All reproduced material is properly cited and acknowledged in the manuscript.

## Data Availability

The data that support the findings of this study are available from the corresponding author upon reasonable request. Data sharing is subject to ethical and legal considerations.
